# Changes in the treatment rate of patients newly diagnosed with stage IV cancer near the end of life from 2012 to 2017 in Korea

**DOI:** 10.4178/epih.e2023021

**Published:** 2023-02-14

**Authors:** Kyuwoong Kim, Hyun Jung Jho, So Jung Park, Bohyun Park, Jin Young Choi

**Affiliations:** 1 National Hospice Center, National Cancer Control Institute, Goyang, Korea; 2 Department of Hospice Palliative Service, National Cancer Center, Goyang, Korea

**Keywords:** Neoplasms, Therapeutics, Death, Database

## Abstract

**OBJECTIVES:**

This study aimed to evaluate changes in the cancer treatment rate among patients newly diagnosed with stage IV cancer using socio-demographic and clinical subgroups in a nationwide cohort of Korean patients.

**METHODS:**

This retrospective, national-level study used the Korea Central Cancer Registry (KCCR), which is linked to the National Health Insurance Service (NHIS) database, from January 1, 2012 to December 31, 2017. The records of patients newly diagnosed with stage IV of the 5 cancers with the highest cancer-related mortality rate were identified to analyze changes in the treatment rate. The main outcome examined in this study was the change in the cancer treatment rate between 2012 and 2017, as measured using the annual percent change (APC).

**RESULTS:**

A total of 106,082 patients with newly diagnosed gastric, colorectal, liver, pancreatic, and lung cancers at the end of life (EoL) were identified from the KCCR-NHIS database. Of these patients, 76,533 (72.1%) received cancer treatment. Over the study period (2012-2017), the proportion of patients who received cancer treatment at EoL decreased by 8.3%, with an APC of -2.1% (95% confidence interval, -2.6 to -1.6). This declining trend of cancer treatment among patients with advanced cancer stage at EoL was consistent among socio-demographic and clinical subgroups.

**CONCLUSIONS:**

The proportion of untreated patients with stage IV cancer is increasing in the Korea. For patients who are not undergoing standard cancer treatment near EoL, an alternative care plan, such as early palliative care, should be considered.

## GRAPHICAL ABSTRACT

[Fig f5-epih-45-e2023021]

## INTRODUCTION

Cancer, followed by cardiovascular disease, is the leading cause of mortality worldwide, resulting in approximately 10 million deaths in 2020 [1]. In the recent analysis of the Prospective Urban Rural Epidemiology study, hospital admission and death rates from cancer in high-income countries were nearly 2-fold to 3-fold higher than those in middle-income and low-income countries across 5 continents [2]. Based on current trends in cancer incidence and mortality, the global burden of cancer is expected to double by 2070 if additional cancer control plans are not implemented worldwide [3]. Despite the use of traditional and newly emerging health-related risk factors [4-11], the cancer stage at diagnosis continues to be a strong predictor of cancer mortality [12-14].

Most randomized controlled trials and observational studies have demonstrated improved survival outcomes among patients with advanced cancer stage who received conventional or novel treatments compared to those who did not [15,16], but these studies have not extensively addressed those who were not treated for cancer near the end of life (EoL) [17]. Currently, there is a paucity of data on overall trends of cancer treatment among patients newly diagnosed with advanced-stage cancer near EoL, which is a proxy for the accessibility of standard or alternative cancer care, including hospice and palliative care (HPC) services [18-20]. Generally, the main needs of cancer patients at their EoL are multifactorial, ranging from psychiatric care to social support from clinicians and caregivers [21,22]. Moreover, even though aggressive treatment for patients with advanced cancer is relatively affordable under a single-payer healthcare system in Korea compared to privatized healthcare systems elsewhere, there are limited data on recent trends in the treatment rate and its differences between socio-demographic and clinical subgroups [23,24].

This study aimed to compare up-to-date, large-scale, administrative data on the aforementioned trends among patients with stage IV cancer near their EoL in Korea between socio-demographic and clinical subgroups to evaluate whether treatment trends are changing.

## MATERIALS AND METHODS

### Study design

We conducted a population-based, retrospective cohort study using nationwide data from the Korea Central Cancer Registry linked to the National Health Insurance Service (KCCR-NHIS) from January 1, 2012 to December 31, 2017 to investigate trends in cancer treatment near EoL by cancer types, socio-demographic factors, and clinical characteristics. We also evaluated trends in types of cancer treatment among patients who received treatment.

### Data source

We collected data on the 5 non-sex-specific cancers with the highest cancer-related mortality rates from the KCCR, which is a nationwide cancer registry that is extensively used for monitoring cancer incidence, mortality, and survival and was established by the Ministry of Health and Welfare and operated by the National Cancer Center in Korea. After identifying newly diagnosed cases of stage IV gastric, colorectal, liver, pancreatic, and lung cancers using the Surveillance, Epidemiology, and End Results Program (SEER) staging guidelines in KCCR, we linked the data of these patients to the NHIS to obtain information on the socio-demographic factors and clinical characteristics from the insurance eligibility and medical claims records of the NHIS, respectively. Briefly, the NHIS is the single insurer for health insurance in Korea, with a coverage rate of approximately 97%. The NHIS provides a variety of databases, including those for insurance premiums, which serve as a proxy for income status, and pharmaceutical prescription claims, for research purposes, especially for epidemiological studies and health policymaking. The details of the KCCR and NHIS databases have been described in previous studies [25-27].

### Population

**Figure 1.**
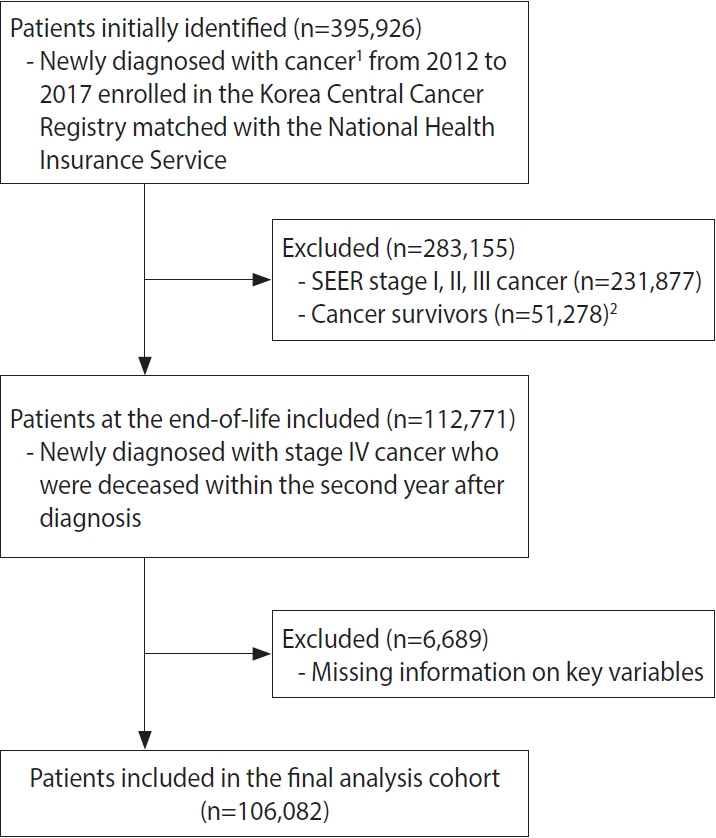
Study population flowchart. SEER, Surveillance, Epidemiology, and End Results. ^1^Non-sex-specific cancers (gastric, colorectal, liver, pancreatic, and lung cancers) with the highest mortality rate in Korea. ^2^Patients who survived after the second year postdiagnosis.

The study population included adult patients aged ≥ 20 years enrolled in the database between 2012 and 2017 with records of newly diagnosed stage IV gastric, colorectal, liver, pancreas, or lung cancers, which are the leading causes of cancer-related mortality for both male and female in Korea [26]. Cancer types in the KCCR database were identified using the International Classification of Diseases for Oncology, 3rd edition. We excluded patients with cancers identified in the medical claims records in any site prior to the diagnosis year according to the International Classification of Diseases, 10th revision (C00-C97). To explore treatment trends near EoL, we only included patients who died within 2 years after the initial diagnosis (n= 106,082). The selection criteria for the study population are shown in Figure 1.

### Measures

Cancer treatment was measured using the NHIS records pertaining to medical claims for monotherapy (surgery only, chemotherapy only, and radiation therapy only) and combination therapy (2 or more therapies). We retrieved the medical claims records for cancer treatment from the initial diagnosis to death among the patients enrolled. For the primary outcome, patients treated for cancer were defined as those who had received any type of treatment during the study period, whereas those who had no treatment were defined as untreated. Socio-demographic and clinical variables included age (< 70 and ≥ 70 years of age considered to indicate non-elderly and elderly status, respectively), sex (male or female), place of residence (categorized with administrative codes as capital, metropolitan, or rural), insurance type (employee insured or self-employed insured), insurance premium (grouped into quartile representing income status), and Charlson comorbidity index (CCI, calculated from the medical claims records prior to the first-ever cancer diagnosis).

### Statistical analysis

The socio-demographic and clinical characteristics of treated and untreated patients are presented as numbers and percentages for each variable. The chi-square test was used to compute p-values between the 2 aforementioned populations for each characteristic. To assess the yearly treatment rate, we computed the proportion of patients who were treated for stage IV cancer as the crude rate (CR) per 1,000 patients. We also calculated the CR per 1,000 people for the treatment and non-treatment rates as stratified according to cancer sites (gastric, colorectal, liver, pancreas, and lung) and socio-demographic (age, sex, place of residence, insurance type, and insurance premium) and clinical characteristics (CCI). Among the treated patients, the crude proportions for types of treatment near EoL were calculated. To estimate the changing trends in these treatment patterns from 2012 to 2017, we computed annual percent changes (APCs) with 95% confidence intervals (CIs).

As a sensitivity analysis, we additionally computed APCs with 95% CIs for the cancer treatment rate derived from the age-standardized rate (ASR) using the population aged more than 20 years in 2012 as the standard population. Statistical significance was 2-sided, and a p-value < 0.05 was considered statistically significant. Data collection and analyses of patient characteristics and treatment patterns each year were conducted using SAS version 9.4 (SAS Institute Inc., Cary, NC, USA). The Joinpoint Regression Program version 4.9.0.1 from the National Cancer Institute (Rockville, MD, USA) was used to calculate APCs and their 95% CIs for the trend analysis.

### Ethics statement

This study was approved by the Institutional Review Board of the National Cancer Center (approval No. NCC2020-0064). There was no requirement to obtain verbal or written consent from patients because the KCCR-NHIS database is anonymized according to the Personal Information Protection Act.

## RESULTS

### Study population

In total, we identified 106,082 patients newly diagnosed with stage IV cancer near EoL from 2012 to 2017, among whom 76,533 (72.1%) were treated and 29,549 (27.9%) were untreated following the diagnosis. Most untreated patients were adults aged ≥ 70 years (71.9%), whereas the proportion of older adults was significantly lower among treated patients than among untreated patients (43.1%). Compared with untreated patients, treated patients more frequently resided in the capital city or a metropolitan area. Lung cancer was the most common type of cancer in both groups (45.4 and 40.6% in the treated and untreated groups, respectively). Among treated patients, chemotherapy alone (57.8%) and combination therapy (37.2%) were the most prevalent types of cancer treatment. Table 1 shows the differences in the socio-demographic and clinical characteristics between treated and untreated patients.

### Trends in cancer treatment near end of life

**Figure 2.**
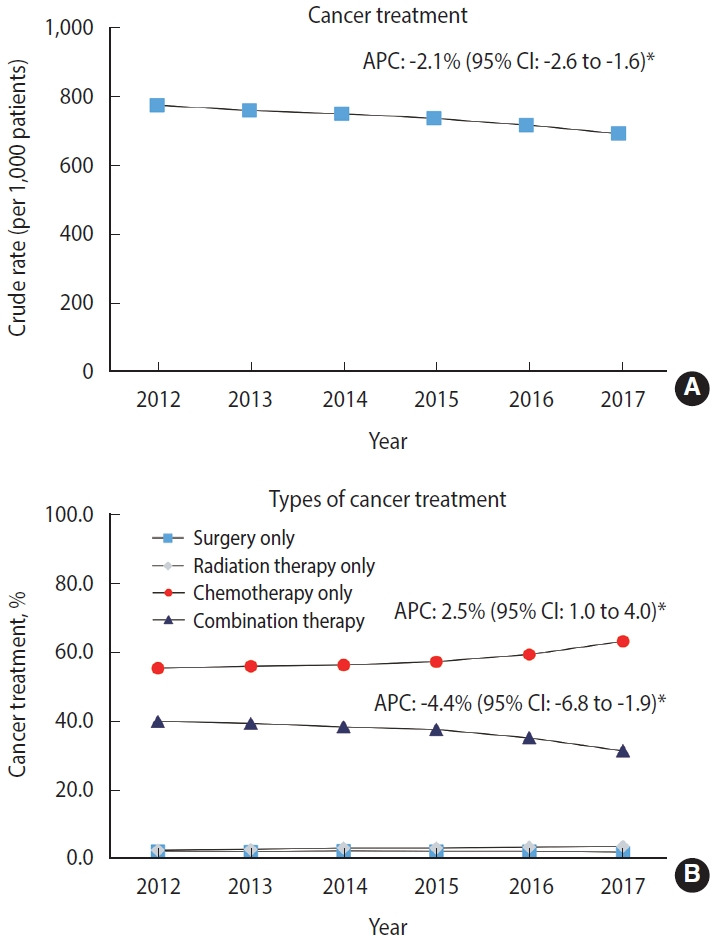
Trends in the cancer treatment rate among patients newly diagnosed with stage IV cancer near the end of life between 2012 and 2017. Non-sex-specific cancer sites included gastric, colorectal, liver, pancreas, and lung based on the cancers with the highest cancer-related mortality rate in Korea. APC, annual percent change; CI, confidence interval. APC of surgery only: -1.9% (95% CI, -6.1 to 2.5). APC of chemotherapy only: 7.7% (95% CI, 5.4 to 10.0). ^*^p<0.05.

Figure 2A shows the CR per 1,000 patients for the trend in cancer treatment in patients newly diagnosed with stage IV cancer near EoL. In 2012, 13,763 (77.7%) patients were treated (CR, 777.6; 95% CI, 764.5 to 790.6), but this proportion decreased by 8.3% in 2017 (CR, 694.8; 95% CI, 681.3 to 708.2). From 2012 to 2017, cancer treatment among these patients significantly decreased, with an APC of -2.1% (95% CI, -2.6 to -1.6; p< 0.05). Figure 2B shows trends in the types of cancer treatment among patients who were treated with either monotherapy or combination therapy. In 2012, 5,498 of 13,763 (39.9%) patients received combination therapy, but this proportion significantly decreased by 8.6% in 2017 with an APC of -4.4% (95% CI, -6.8 to -1.9; p< 0.05). Meanwhile, the proportion of those who received chemotherapy alone significantly increased from 2012 to 2017 (APC, 2.5%; 95% CI, 1.0 to 4.0; p< 0.05). Details of the cancer treatment in each year between 2012 and 2017 are presented in Supplementary Materials 1 and 2. The sensitivity analysis showed that the decreasing trend in cancer treatment remained consistent when the CR was converted to the ASR (APC, -3.4%; 95% CI, -4.5 to -2.3; p< 0.05) (Supplementary Material 3).

### Discrepancies in treatment trend near end of life between cancer sites

**Figure 3.**
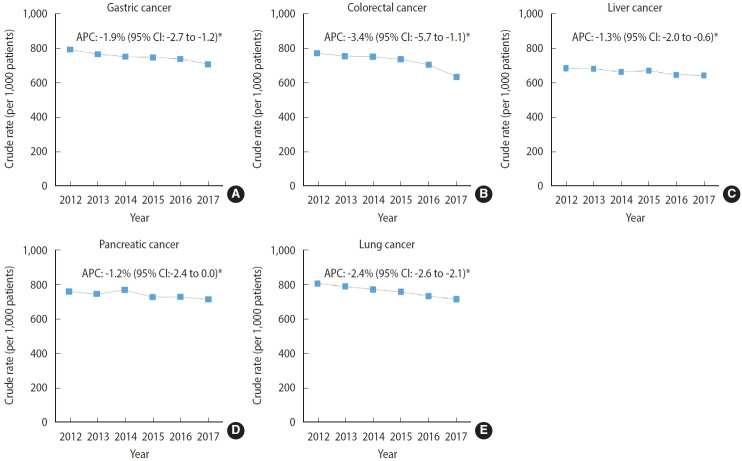
Trends in the site-specific cancer (A: gastric, B: colorectal, C: liver,D: pancreas, and E: lung) treatment rate among patients newly diagnosed with stage IV cancer near the end of life between 2012-2017. APC, annual percent change; CI, confidence interval. ^*^p<0.05.

Figure 3 shows the site-specific rates for cancer treatment near EoL among the cancers with the highest cancer-related mortality rates in male and female from 2012 to 2017. The magnitude of the decreasing trend in the cancer treatment rate was the highest among patients with colorectal cancer (APC, -3.4%; 95% CI, -5.7 to -1.1; p< 0.05), followed by those with lung cancer (APC, -2.4%; 95% CI, -2.6 to -2.1; p< 0.05). The APCs for cancer treatment in other sites also showed significantly decreasing trends in patients with gastric cancer (APC, -1.9%; 95% CI, -2.7 to -1.2; p< 0.05) and liver cancer (APC, -1.3%; 95% CI, -2.0 to -0.6; p< 0.05), but a non-significant trend was found in patients with pancreatic cancer (APC, -1.2%; 95% CI, -2.4 to 0.0; p< 0.05). Supplementary Material 4 shows the details on treatment trends by cancer sites between 2012 and 2017.

### Treatment trends by socio-demographic and clinical characteristics

**Figure 4.**
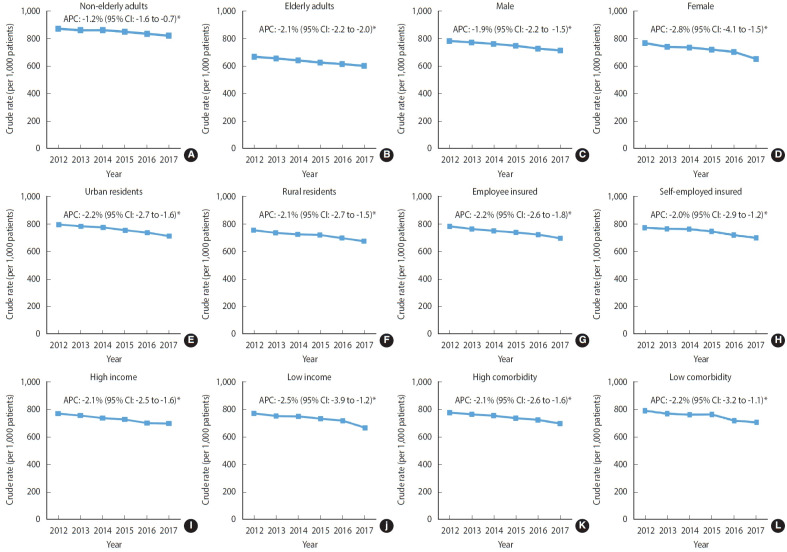
Trends in the cancer treatment rate according to the socio-demographic and clinical characteristics of patients newly diagnosed with stage IV cancer near the end of life between 2012 and 2017 (A) non-elderly adults, (B) elderly adults, (C) male, (D) female, (E) urban residents, (F) rural resident, (G) employee insured, (H) self-employed insured, (I) high income, (J) low income, (K) high comorbidity, and (L) low comorbidity . Non-sex-specific cancer sites included gastric, colorectal, liver, pancreas, and lung based on the cancers with the highest cancer-related mortality rates in Korea. APC, annual percent change; CI, confidence interval. ^*^p<0.05.

As shown in Figure 4, the decreasing trend in the cancer treatment rate was similar across all socio-demographic and clinical characteristics from 2012 to 2017. Patients aged ≥ 70 years showed a more drastic decrease in the cancer treatment rate (APC, -2.1%; 95% CI, -2.2 to -2.0; p< 0.05) than did those aged ≤ 70 years (APC, -1.2%; 95% CI, -1.6 to -0.7; p< 0.05). Compared with male patients (APC, -1.9%; 95% CI, -2.2 to -1.5; p< 0.05), female patients showed a steeper decline in the rate of cancer treatment over the time period (APC, -2.8%; 95% CI, -4.1 to -1.5; p< 0.05). Decreasing trends of similar magnitude were observed among patients with different residential areas, insurance type, insurance premiums (indicator for income status), and comorbid conditions. Supplementary Material 5 lists the proportions and CRs (95% CI) of treated and untreated patients by their characteristics each year between 2012 and 2017.

## DISCUSSION

Among the 106,082 patients who were newly diagnosed with stage IV cancers from 2012 to 2017 near EoL in Korea, the rate of cancer treatment showed a progressive decline, most notably among patients with colorectal and lung cancers. Overall, the decreasing trend in cancer treatment was consistent in patients with different socio-demographic and clinical characteristics. Nonetheless, the proportion of patients who were treated for stage IV cancer was persistently around 70% during the study period, representing that most patients near their EoL were still being treated.

Moreover, the rate of combination therapy declined, and that of chemotherapy alone increased among treated patients throughout the study period. Among those who remained untreated for stage IV cancer near their EoL, the awareness of an alternative clinical approach, such as HPC, and socioeconomic barriers to access to treatment might have partially contributed to the declining trend in the treatment rate.

### Comparison to other studies

Few studies have measured changes in treatment patterns and clinical outcomes among patients with cancer, but the results were mixed, and those studies have seldom focused on the comprehensive assessment of the treatment rate among stage IV patients near their EoL over time. Data from the Rotterdam Cancer Registry, which holds information on patients in the southwestern part of the Netherlands, showed a 38% and 6% increase in chemotherapy use and hepatic surgery, respectively, among 3,482 patients diagnosed with stage IV colorectal cancer during the study period (1995-2007) [28]. Similar to the Rotterdam study, our findings also show an increasing trend in the use of chemotherapy for cancer treatment between 2012 and 2017 among patients who were treated for stage IV cancers. However, the Rotterdam study only examined the trend in standard oncology treatment, including surgery and chemotherapy, in patients with advanced-stage colorectal cancer, without considering alternative care options, such as the early utilization of HPC. Furthermore, the Rotterdam study was based on a single Dutch city and did not examine patients with stage IV cancer near their EoL. Considering the substantially high treatment rate and improved survival outcomes reported in the Rotterdam study, patients were more likely to cope with treatment compared to those who were clinically unfit to receive treatment due to overall health or degree of cancer progression.

Consistent with the Rotterdam study, another population-based study conducted among elderly Dutch patients with stage III colon cancer from the Eindhoven Cancer Registry showed an increasing rate of adjuvant chemotherapy between 1995 and 2001 [29]. However, in the Eindhoven study, elderly patients who were female and with low socioeconomic status (SES) and comorbid conditions were less likely to receive this treatment over the study period. In contrast to the Eindhoven study, the results from the current KCCR-NHIS study showed a consistently declining trend in the treatment rate, regardless of SES, including income status and health insurance type, and comorbidities, including the CCI. Moreover, the Eindhoven study used postal codes to estimate SES among elderly patients, rather than directly assessing income or the financial assets of patients. Since SES plays a key role in access to cancer treatment, obtaining accurate measures of SES is critical when examining the changes in cancer treatment trends by socio-demographic subgroups.

Moreover, the differences in the trend of cancer treatment rate among patients with low SES between the Eindhoven study and current KCCR-NHIS study could be partially explained by the enhanced insurance coverage for cancer treatment under a single insurer system with the NHIS in Korea.

### Study implications

The findings of this nationwide cohort study have important implications for public health policy for the management of patients with cancer near their EoL, suggesting that patients and their caregivers should be thoroughly informed about available clinical decisions and expected outcomes for each option. In a non-blinded, randomized, controlled trial of patients newly diagnosed with metastatic non-small-cell lung cancer (NSCLC) in the United States, patients with NSCLC who received early utilization of palliative care had improved quality of life (QoL) and better mood and received less aggressive care, including chemotherapy, within 2 weeks prior to death and without hospice care or admission to the hospice ward 3 days or less prior to death, compared to those who received standard cancer care towards their EoL [30].

Moreover, a recent cost-utility analysis conducted in China suggested that palliative care improved the QoL at EoL and mitigated the economic burden for Chinese patients with advanced cancer [24]. Integrating the evidence from these studies, early HPC, as well as standard oncology care, should be suggested as a viable option for patients whose cancer is unlikely to be cured or successfully managed owing to its advantages. Thus, healthcare professionals and health policymakers should be aware of the potential advantages and disadvantages of both standard oncology care and HPC. Future investigations are warranted to explore whether the decreasing trend of cancer treatment among patients at their EoL is partially attributable to discrepancies in socioeconomic and clinical background among patients, changes in treatment guidelines and health insurance coverage, regional variations in cancer treatment hospitals, and patients’ refusal of life-sustaining treatment.

### Strengths and limitations

To the best of our knowledge, no previous studies have extensively examined recent trends in the treatment rate of patients with stage IV cancers near their EoL by socio-demographic and clinical subgroups using large-scale observational data in Korea, where the NHIS is the single insurer. In fact, most previous studies have focused on patterns of care and survival outcomes of patients with stage IV or earlier stages. Furthermore, this study was conducted with high-quality data on cancer type, diagnosis date, and SEER stage linked to population-based data on the socio-demographic and clinical situation of patients. Therefore, a major strength of our study is that we abstracted information from the nationwide data on cancer patients near their EoL with a high coverage rate in Korea. In this regard, our study is less prone to selection bias than single-center studies assessing a registry with a low coverage rate. Nevertheless, this study has a few limitations. There was no detailed information on the types of chemotherapy, including direct DNA-interacting agents, targeted therapy, immunotherapy, hormone therapy, and so on, in our dataset. Future studies should assess trends in chemotherapy use among cancer patients at EoL, taking into account differences in the level of toxicity by types of chemotherapy. Moreover, the data obtained from the KCCR-NHIS database do not contain information on the reason behind the decision not to be treated for stage IV cancer, which would include, for example, a high financial burden pertaining to cancer treatment or a desire not to receive aggressive treatment near EoL. However, almost the entire Korean population is enrolled in the NHIS, and those diagnosed with cancer benefit from a substantially discounted copayment rate after the implementation of the health insurance coverage expansion policy for cancer in 2013. Moreover, our study showed a declining treatment rate for both high-income and low-income patients with stage IV cancers. Thus, it is relatively unlikely that the declining trend of cancer treatment near EoL is attributable to cancer-related financial burdens.

In conclusion, in this nationwide cohort study of KCCR-NHIS data from 2012 to 2017, there was a significantly decreasing trend in the treatment rate of patients diagnosed with stage IV cancers near their EoL, though most patients still received cancer treatment. On the basis of the currently available evidence, alternative clinical choices, such as the early utilization of HPC, may thoroughly improve QoL and reduce aggressive care for patients with advanced-stage cancer at EoL.

## Figures and Tables

**Figure f5-epih-45-e2023021:**
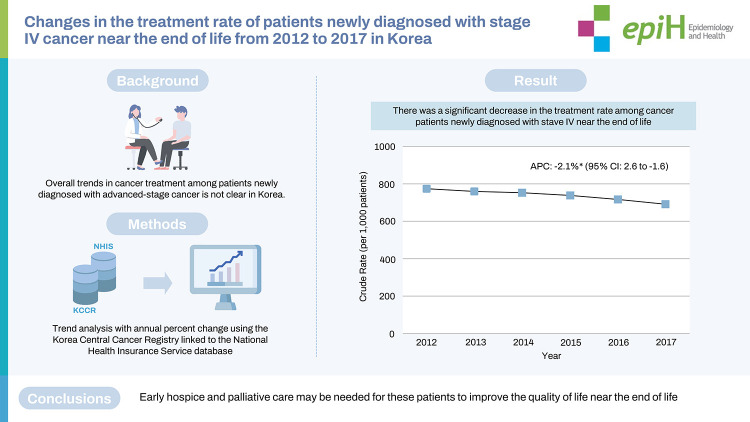


**Table 1.** Socioeconomic and clinical characteristics of the patients newly diagnosed with stage IV cancer with and without cancer treatment identified in the Korea Central Cancer Registry (KCCR) linked to the National Health Insurance Service database from 2012 to 2017

Characteristics			Patients newly diagnosed with stage IV cancer^1^		
			Treated (n=76,533)^2^	Untreated (n=29,549)^3^	p-value^4^
Age (yr)^5^					
	20-29		210 (0.3)	21 (0.1)	<0.001
	30-39		1,576 (2.1)	193 (0.7)	
	40-49		5,581 (7.3)	821 (3.1)	
	50-59		15,164 (19.8)	2,466 (9.3)	
	60-69		21,022 (27.5)	3,962 (14.9)	
	≥70		32,980 (43.1)	19,086 (71.9)	
Sex					
	Male		52,788 (69.0)	17,449 (65.7)	<0.001
	Female		23,745 (31.0)	9,100 (34.3)	
Place of residence					
	Capital city		14,164 (18.5)	3,981 (15.0)	<0.001
	Metropolitan		33,879 (44.3)	11,281 (42.5)	
	Rural (city/town)		28,490 (37.2)	11,287 (42.5)	
Insurance type					
	Employee insured		49,933 (65.2)	17,523 (66.0)	0.026
	Self-employed insured		26,600 (34.8)	9,026 (34.0)	
Insurance premium^6^					
	Quartile 1		14,854 (19.4)	5,478 (20.6)	<0.001
	Quartile 2		14,850 (19.4)	4,726 (17.8)	
	Quartile 3		18,655 (24.4)	5,915 (22.3)	
	Quartile 4		28,174 (36.8)	10,430 (39.3)	
Cancer type					
	Gastric		11,875 (15.5)	3,890 (14.7)	<0.001
	Colorectal		11,950 (15.6)	4,369 (16.5)	
	Liver		7,911 (10.3)	3,982 (15.0)	
	Pancreas		10,051 (13.1)	3,540 (13.3)	
	Lung		34,746 (45.4)	10,768 (40.6)	
Treatment for cancer					
	Monotherapy				
		Surgery	1,599 (2.1)	-	
		Chemotherapy	44,222 (57.8)	-	
		Radiation therapy	2,250 (2.9)	-	
	Combination therapy		28,462 (37.2)	-	
Charlson comorbidity index^7^					
	0		4,900 (6.4)	1,776 (6.7)	<0.001
	1		13,144 (17.2)	4,095 (15.4)	
	≥2		58,489 (76.4)	20,678 (77.9)	

Values are presented as number (%).

1 Five non-sex-specific cancers with the highest cancer mortality based on the KCCR report.

2 Patients newly diagnosed with stage IV cancer who underwent surgery, chemotherapy, radiotherapy, or combination treatment prior to death.

3 Patients newly diagnosed with stage IV cancer who did not receive any type of treatment for cancer prior to death.

4 Computed with the chi-square test.

5 Age at the time of first-ever stage IV cancer diagnosis.

6 Proxy for individual-level income status.

7 Without accounting for assigned weights in comorbid conditions related to cancer.

## SUPPLEMENTARY MATERIALS

Supplementary Material 1. Trends in cancer treatment among patients with newly diagnosed stage IV cancers identified in the Korea Central Cancer Registry linked to the National Health Insurance Service database from 2012 to 2017

Supplementary Material 2. Trends in treatment types among patients treated for newly diagnosed stage IV cancers identified in the Korea Central Cancer Registry linked to the National Health Insurance Service database from 2012 to 2017

Supplementary Material 3. Trend in the cancer treatment rate (age-standardized rate) among patients with newly diagnosed stage IV cancers near the end of life between 2012 and 2017

Supplementary Material 4. Trends in treatment by cancer sites among patients for newly diagnosed stage IV cancers identified in the Korea Central Cancer Registry linked to the National Health Insurance Service database from 2012 to 2017

Supplementary Material 5. Trends in cancer treatment by socioeconomic factors and comorbid conditions among patients with newly diagnosed stage IV cancers identified in the Korea Central Cancer Registry linked to the National Health Insurance Service database from 2012 to 2017
