# Supplementary material for: Changes in the treatment rate of patients newly diagnosed with stage IV cancer near the end of life from 2012 to 2017 in Korea
Source: Epidemiol Health. 2023 Feb 14;45:e2023021. doi: 10.4178/epih.e2023021 (PMC10266927; doi:10.4178/epih.e2023021)
Supplement: Supplementary Material 2. — Trends in treatment types among patients treated for newly diagnosed stage IV cancers identified in the Korea Central Cancer Registry linked to the National Health Insurance Service database from 2012 to 2017 [file epih-45-e2023021-Supplementary-2.docx]

**Supplementary Material 2.** Trends in treatment types among patients treated for newly diagnosed stage IV cancers identified in the Korea Central Cancer Registry linked to the National Health Insurance Service database from 2012 to 2017

|  | **Year** | | | | | | **Overall Trend** |
| --- | --- | --- | --- | --- | --- | --- | --- |
|  | **2012** | **2013** | **2014** | **2015** | **2016** | **2017** | **APC (95% CI)** |
| Monotherapy |  |  |  |  |  |  |  |
| Surgery | 294 (2.1) | 270 (2.0) | 310 (2.2) | 279 (2.1) | 259 (2.1) | 187 (1.8) | -1.9 (-6.1 to 2.5) |
| Chemotherapy | 7,638 (55.5) | 7,637 (56.1) | 7,783 (56.4) | 7,455 (57.4) | 7,211 (59.5) | 6,498 (63.4) | 2.5 (1.0 to 4.0)^*^ |
| Radiation therapy | 333 (2.4) | 355 (2.6) | 417 (3.0) | 389 (3.0) | 400 (3.3) | 356 (3.5) | 7.7 (5.4 to 10.0)^**^ |
| Combination therapy^a^ | 5,498 (39.9) | 5,350 (39.3) | 5,280 (38.3) | 4,868 (37.5) | 4,252 (35.1) | 3,214 (31.3) | -4.4 (-6.8 to -1.9)^**^ |

NOTE: Data presented above include patients with five types of non-sex-specific cancer (gastric, colorectal, liver, pancreas, and lung) who were newly diagnosed with stage IV cancer from 2012 to 2017 and deceased between 2012 and 2018.Selection criteria for these non-sex-specific cancers was cancer mortality from the KCCR report. All values are presented as no.(%) per annum except for change in trend (APC with 95% CI)

Abbreviations: no., number; APC, annual percent change; KCCR, Korea Central Cancer Registry

^a^Includes more than one treatment method for cancer treatment for the five types of cancers listed above

**p*-value <0.05; ***p*-value <0.01
