# Supplementary material for: Changes in the treatment rate of patients newly diagnosed with stage IV cancer near the end of life from 2012 to 2017 in Korea
Source: Epidemiol Health. 2023 Feb 14;45:e2023021. doi: 10.4178/epih.e2023021 (PMC10266927; doi:10.4178/epih.e2023021)
Supplement: Supplementary Material 3. — Trend in the cancer treatment rate (age-standardized rate) among patients with newly diagnosed stage IV cancers near the end of life between 2012 and 2017 [file epih-45-e2023021-Supplementary-3.docx]

**Supplementary Material 3.** Trend in the cancer treatment rate (age-standardized rate) among patients with newly diagnosed stage IV cancers near the end of life between 2012 and 2017


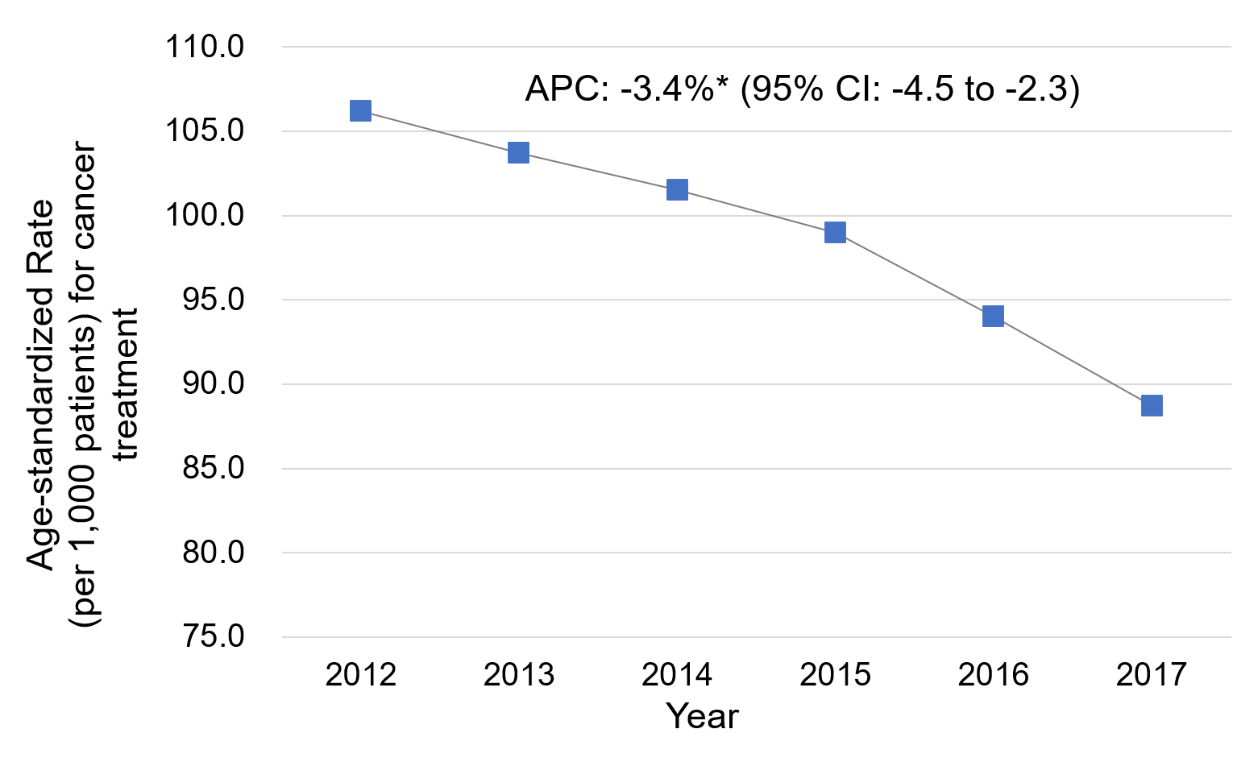


NOTE: The following cancer types were included: gastric cancer, colorectal cancer, liver cancer, pancreatic cancer, and lung cancer. Age-standardized rate was computed by standardizing age distribution of the Korean population in 2012

Acronym: APC, annual percent change

*p<0.05
