# Supplementary material for: Changes in the treatment rate of patients newly diagnosed with stage IV cancer near the end of life from 2012 to 2017 in Korea
Source: Epidemiol Health. 2023 Feb 14;45:e2023021. doi: 10.4178/epih.e2023021 (PMC10266927; doi:10.4178/epih.e2023021)
Supplement: Supplementary Material 4. — Trends in treatment by cancer sites among patients for newly diagnosed stage IV cancers identified in the Korea Central Cancer Registry linked to the National Health Insurance Service database from 2012 to 2017 [file epih-45-e2023021-Supplementary-4.docx]

**Supplementary Material 4.** Trends in treatment by cancer sites among patients for newly diagnosed stage IV cancers identified in the Korea Central Cancer Registry linked to the National Health Insurance Service database from 2012 to 2017

|  | **Year** | | | | | | **Overall Trend** |
| --- | --- | --- | --- | --- | --- | --- | --- |
|  | **2012** | **2013** | **2014** | **2015** | **2016** | **2017** | **APC (95% CI)** |
| Gastric |  |  |  |  |  |  |  |
| Treated^a^ |  |  |  |  |  |  |  |
| No. (%) | 2,329 (79.3) | 2,100 (76.6) | 2,138 (75.2) | 1,951 (74.8) | 1,866 (73.8) | 1,491 (70.8) | -1.9 (-2.6 to -1.2)^**^ |
| CR (95% CI) | 793 (760.8-825.2) | 766.1 (733.4-798.9) | 752 (720.1-783.9) | 747.5 (714.3-780.7) | 738.4 (704.9-771.9) | 707.6 (671.7-743.6) | -1.9 (-2.7 to -1.2)^**^ |
| Untreated^b^ |  |  |  |  |  |  |  |
| No. (%) | 608 (20.7) | 641 (23.4) | 705 (24.8) | 659 (25.2) | 661 (26.2) | 616 (29.2) | 6.1 (3.8 to 8.5)^**^ |
| CR (95% CI) | 207 (190.6-223.5) | 233.9 (215.8-252) | 248 (229.7-266.3) | 252.5 (233.2-271.8) | 261.6 (241.6-281.5) | 292.4 (269.3-315.4) | 6.1 (3.8 to 8.5)^**^ |
| Colorectal |  |  |  |  |  |  |  |
| Treated^a^ |  |  |  |  |  |  |  |
| No. (%) | 2,359 (77.1) | 2,265 (75.4) | 2,241 (75.1) | 2,103 (73.6) | 1,819 (70.5) | 1,163 (63.3) | -3.4 (-5.7 to -1.1)^*^ |
| CR (95% CI) | 771.2 (740-802.3) | 754.2 (723.2-785.3) | 751.3 (720.2-782.4) | 736.1 (704.6-767.5) | 705 (672.6-737.4) | 633.1 (596.7-669.5) | -3.4 (-5.7 to -1.1)^*^ |
| Untreated^b^ |  |  |  |  |  |  |  |
| No. (%) | 700 (22.9) | 738 (24.6) | 742 (24.9) | 754 (26.4) | 761 (29.5) | 674 (36.7) | 8.8 (3.9 to 13.0)^**^ |
| CR (95% CI) | 228.8 (211.9-245.8) | 245.8 (228-263.5) | 248.7 (230.8-266.6) | 263.9 (245.1-282.8) | 295 (274-315.9) | 366.9 (339.2-394.6) | 8.8 (3.9 to 14.0)^**^ |
| Liver |  |  |  |  |  |  |  |
| Treated^a^ |  |  |  |  |  |  |  |
| No. (%) | 1,481 (68.4) | 1,415 (68.1) | 1,368 (66.2) | 1,291 (67.0) | 1,216 (64.5) | 1,140 (64.3) | -1.3 (-2.0 to -0.6)^**^ |
| CR (95% CI) | 684.1 (649.2-718.9) | 681.3 (645.8-716.8) | 662.5 (627.4-697.6) | 670 (633.4-706.5) | 645.1 (608.8-681.4) | 642.6 (605.3-679.9) | -1.3 (-2.0 to -0.6)^**^ |
| Untreated^b^ |  |  |  |  |  |  |  |
| No. (%) | 684 (31.6) | 662 (31.9) | 697 (33.8) | 636 (33.0) | 669 (35.5) | 634 (35.7) | 2.6 (1.2 to 4.1)^**^ |
| CR (95% CI) | 315.9 (292.3-339.6) | 318.7 (294.4-343) | 337.5 (312.5-362.6) | 330 (304.4-355.7) | 354.9 (328-381.8) | 357.4 (329.6-385.2) | 2.6 (1.3 to 4.1)^**^ |
| Pancreas |  |  |  |  |  |  |  |
| Treated^a^ |  |  |  |  |  |  |  |
| No. (%) | 1,562 (75.9) | 1,484 (74.5) | 1,785 (76.9) | 1,752 (72.7) | 1,786 (72.8) | 1,682 (71.4) | -1.2 (-2.4 to 0.0) |
| CR (95% CI) | 759.4 (721.7-797) | 745.4 (707.4-783.3) | 768.7 (733.1-804.4) | 726.7 (692.6-760.7) | 728.1 (694.3-761.9) | 713.6 (679.5-747.7) | -1.2 (-2.4 to 0.0) |
| Untreated^b^ |  |  |  |  |  |  |  |
| No. (%) | 495 (24.1) | 507 (25.5) | 537 (23.1) | 659 (27.3) | 667 (27.2) | 675 (28.6) | 3.5 (-0.2 to 7.4) |
| CR (95% CI) | 240.6 (219.4-261.8) | 254.6 (232.5-276.8) | 231.3 (211.7-250.8) | 273.3 (252.5-294.2) | 271.9 (251.3-292.5) | 286.4 (264.8-308) | 3.6 (-0.1 to 7.4) |
| Lung |  |  |  |  |  |  |  |
| Treated^a^ |  |  |  |  |  |  |  |
| No. (%) | 6,032 (80.6) | 6,348 (78.9) | 6,258 (77.2) | 5,894 (75.7) | 5,435 (73.4) | 4,779 (71.5) | -2.4 (-2.6 to -2.1)^***^ |
| CR (95% CI) | 806.2 (785.9-826.5) | 788.6 (769.2-808) | 771.9 (752.8-791.1) | 757.5 (738.1-776.8) | 733.6 (714.1-753.1) | 714.9 (694.6-735.2) | -2.4 (-2.6 to -2.1)^***^ |
| Untreated^b^ |  |  |  |  |  |  |  |
| No. (%) | 1,450 (19.4) | 1,702 (21.1) | 1,849 (22.8) | 1,887 (24.3) | 1,974 (26.6) | 1,906 (28.5) | 8.0 (7.5 to 8.4)^***^ |
| CR (95% CI) | 193.8 (183.8-203.8) | 211.4 (201.4-221.5) | 228.1 (217.7-238.5) | 242.5 (231.6-253.5) | 266.4 (254.7-278.2) | 285.1 (272.3-297.9) | 8.0 (7.4 to 8.4)^***^ |

NOTE: Data presented above include patients with five types of non-sex-specific cancer (gastric, colorectal, liver, pancreas, and lung) who were newly diagnosed with stage IV cancer from 2012 to 2017 and deceased between 2012 and 2018.Selection criteria for these non-sex-specific cancers was cancer mortality from the KCCR report. All values are presented as no.(%) per annum except for change in trend (APC with 95% CI)
^a^Patients with newly diagnosed stage IV cancer who underwent surgery, chemotherapy, radiotherapy or combination treatment prior to death

^b^Patients with newly diagnosed stage IV cancer who did not receive any type of treatment for cancer prior to death

^c^Crude rate is presented as number of patients per 1,000 population

Abbreviations: no., number; CR, crude rate; CI, confidence interval; APC, annual percent change; KCCR, Korea Central Cancer Registry

**p*-value <0.05; ***p*-value <0.01; ****p*-value<0.001
