# Supplementary material for: Changes in the treatment rate of patients newly diagnosed with stage IV cancer near the end of life from 2012 to 2017 in Korea
Source: Epidemiol Health. 2023 Feb 14;45:e2023021. doi: 10.4178/epih.e2023021 (PMC10266927; doi:10.4178/epih.e2023021)
Supplement: Supplementary Material 5. — Trends in cancer treatment by socioeconomic factors and comorbid conditions among patients with newly diagnosed stage IV cancers identified in the Korea Central Cancer Registry linked to the National Health Insurance Service database from 2012 to 2017 [file epih-45-e2023021-Supplementary-5.docx]

**Supplementary Material 5.** Trends in cancer treatment by socioeconomic factors and comorbid conditions among patients with newly diagnosed stage IV cancers identified in the Korea Central Cancer Registry linked to the National Health Insurance Service database from 2012 to 2017

|  | **Year** | | | | | | **Overall trend** |
| --- | --- | --- | --- | --- | --- | --- | --- |
|  | **2012** | **2013** | **2014** | **2015** | **2016** | **2017** | **APC (95% CI)** |
| Non-elderly^c^ |  |  |  |  |  |  |  |
| Treated^a^ |  |  |  |  |  |  |  |
| No. (%) | 8,281 (87.3) | 7,955 (86.3) | 7,963 (86.3) | 7,531 (85.1) | 6,670 (83.6) | 5,153 (82.3) | -1.1 (-1.5 to -0.8)^*^ |
| CR (95% CI) | 873.2 (854.4-892.1) | 862.7 (843.7-881.7) | 863.2 (844.2-882.2) | 851.2 (831.9-870.4) | 836.4 (816.3-856.4) | 822.6 (800.2-845.1) | -1.2 (-1.6 to -0.7)^*^ |
| Untreated^b^ |  |  |  |  |  |  |  |
| No. (%) | 1,202 (12.7) | 1,266 (13.7) | 1,262 (13.7) | 1,317 (14.9) | 1,305 (16.4) | 1,111 (17.7) | 6.7 (4.7 to 8.8)^*^ |
| CR (95% CI) | 126.8 (119.6-133.9) | 137.3 (129.7-144.9) | 136.8 (129.3-144.3) | 148.8 (140.8-156.9) | 163.6 (154.8-172.5) | 177.4 (166.9-187.8) | 6.8 (4.6 to 8.9)^*^ |
| Elderly^d^ |  |  |  |  |  |  |  |
| Treated^a^ |  |  |  |  |  |  |  |
| No. (%) | 5,482 (66.7) | 5,657 (65.5) | 5,827 (64.1) | 5,460 (62.5) | 5,452 (61.4) | 5,102 (60.1) | -2.1 (-2.2 to -2.0)^*^ |
| CR (95% CI) | 667.2 (649.5-684.8) | 654.7 (637.6-671.7) | 640.7 (624.2-657.1) | 624.9 (608.3-641.4) | 614 (597.7-630.3) | 600.5 (584-617) | -2.1 (-2.2 to -2.0)^*^ |
| Untreated^b^ |  |  |  |  |  |  |  |
| No. (%) | 2,735 (33.3) | 2,984 (34.5) | 3,268 (35.9) | 3,278 (37.5) | 3,427 (38.6) | 3,394 (39.9) | 3.7 (3.4 to 4.0)^*^ |
| CR (95% CI) | 332.8 (320.4-345.3) | 345.3 (332.9-357.7) | 359.3 (347-371.6) | 375.1 (362.3-388) | 386 (373-398.9) | 399.5 (386-412.9) | 3.8 (3.5 to 4.1)^*^ |
| Male |  |  |  |  |  |  |  |
| Treated^a^ |  |  |  |  |  |  |  |
| No. (%) | 9,353 (78.2) | 9,371 (77.2) | 9,452 (76.1) | 8,984 (74.7) | 8,310 (72.7) | 7,318 (71.4) | -1.9 (-2.2 to -1.5)^*^ |
| CR (95% CI) | 782.1 (766.2-797.9) | 772 (756.3-787.6) | 760.8 (745.4-776.1) | 747.2 (731.7-762.6) | 726.6 (711-742.2) | 713.7 (697.3-730) | -1.9 (-2.2 to -1.5)^*^ |
| Untreated^b^ |  |  |  |  |  |  |  |
| No. (%) | 2,606 (21.8) | 2,768 (22.8) | 2,972 (23.9) | 3,040 (25.3) | 3,127 (27.3) | 2,936 (28.6) | 5.7 (5.0 to 6.5)^*^ |
| CR (95% CI) | 217.9 (209.5-226.3) | 228 (219.5-236.5) | 239.2 (230.6-247.8) | 252.8 (243.8-261.8) | 273.4 (263.8-283) | 286.3 (276-296.7) | 5.8 (5.0 to 6.5)^*^ |
| Female |  |  |  |  |  |  |  |
| Treated^a^ |  |  |  |  |  |  |  |
| No. (%) | 4,410 (76.8) | 4,241 (74.1) | 4,338 (73.6) | 4,007 (72) | 3,812 (70.4) | 2,937 (65.2) | -2.8 (-4.1 to -1.5)^*^ |
| CR (95% CI) | 768.2 (745.5-790.8) | 741 (718.7-763.3) | 735.8 (713.9-757.6) | 720.4 (698.1-742.7) | 703.7 (681.4-726.1) | 651.8 (628.2-675.4) | -2.8 (-4.1 to -1.5)^*^ |
| Untreated^b^ |  |  |  |  |  |  |  |
| No. (%) | 1,331 (23.2) | 1,482 (25.9) | 1,558 (26.4) | 1,555 (28) | 1,605 (29.6) | 1,569 (34.8) | 7.4 (4.5 to 10.3)^*^ |
| CR (95% CI) | 231.8 (219.4-244.3) | 259 (245.8-272.1) | 264.2 (251.1-277.4) | 279.6 (265.7-293.5) | 296.3 (281.8-310.8) | 348.2 (331-365.4) | 7.4 (4.5 to 10.3)^*^ |
| Urban residents^e^ |  |  |  |  |  |  |  |
| Treated^a^ |  |  |  |  |  |  |  |
| No. (%) | 8,678 (79.4) | 8,490 (78.1) | 8,675 (77.3) | 8,199 (75.2) | 7,628 (73.5) | 6,373 (71) | -2.2 (-2.7 to -1.6)^*^ |
| CR (95% CI) | 793.6 (776.9-810.3) | 780.8 (764.2-797.4) | 772.6 (756.4-788.9) | 751.9 (735.6-768.1) | 734.7 (718.2-751.1) | 709.6 (692.2-727) | -2.2 (-2.7 to -1.6)^*^ |
| Untreated^b^ |  |  |  |  |  |  |  |
| No. (%) | 2,257 (20.6) | 2,383 (21.9) | 2,553 (22.7) | 2,706 (24.8) | 2,755 (26.5) | 2,608 (29) | 7.0 (5.8 to 8.2)^*^ |
| CR (95% CI) | 206.4 (197.9-214.9) | 219.2 (210.4-228) | 227.4 (218.6-236.2) | 248.1 (238.8-257.5) | 265.3 (255.4-275.2) | 290.4 (279.2-301.5) | 7.0 (5.8 to 8.2)^*^ |
| Rural residents^e^ |  |  |  |  |  |  |  |
| Treated^a^ |  |  |  |  |  |  |  |
| No. (%) | 5,085 (75.2) | 5,122 (73.3) | 5,115 (72.1) | 4,792 (71.7) | 4,494 (69.4) | 3,882 (67.2) | -2.1 (-2.6 to -1.5)^*^ |
| CR (95% CI) | 751.7 (731-772.3) | 732.9 (712.8-752.9) | 721.2 (701.5-741) | 717.3 (696.9-737.6) | 694.5 (674.2-714.8) | 671.7 (650.6-692.9) | -2.1 (-2.6 to -1.5)^*^ |
| Untreated^b^ |  |  |  |  |  |  |  |
| No. (%) | 1,680 (24.8) | 1,867 (26.7) | 1,977 (27.9) | 1,889 (28.3) | 1,977 (30.6) | 1,897 (32.8) | 5.3 (4.0 to 6.7)^*^ |
| CR (95% CI) | 248.3 (236.5-260.2) | 267.1 (255-279.3) | 278.8 (266.5-291.1) | 282.7 (270-295.5) | 305.5 (292-319) | 328.3 (313.5-343) | 5.3 (4.0 to 6.7)^*^ |
| Employee-insured |  |  |  |  |  |  |  |
| Treated^a^ |  |  |  |  |  |  |  |
| No. (%) | 8,826 (78) | 8,752 (76) | 8,916 (74.7) | 8,615 (73.5) | 8,038 (71.9) | 6,786 (69.3) | -2.2 (-2.6 to -1.8)^*^ |
| CR (95% CI) | 779.7 (763.4-795.9) | 760.4 (744.5-776.4) | 747.2 (731.7-762.7) | 735.1 (719.5-750.6) | 719 (703.3-734.7) | 692.7 (676.2-709.2) | -2.1 (-2.7 to -1.5)^*^ |
| Untreated^b^ |  |  |  |  |  |  |  |
| No. (%) | 2,494 (22) | 2,757 (24) | 3,016 (25.3) | 3,105 (26.5) | 3,141 (28.1) | 3,010 (30.7) | 6.4 (5.4 to 7.4)^*^ |
| CR (95% CI) | 220.3 (211.7-229) | 239.6 (230.6-248.5) | 252.8 (243.7-261.8) | 264.9 (255.6-274.3) | 281 (271.1-290.8) | 307.3 (296.3-318.2) | 6.5 (5.5 to 7.4)^*^ |
| Self-employed insured |  |  |  |  |  |  |  |
| Treated^a^ |  |  |  |  |  |  |  |
| No. (%) | 4,937 (77.4) | 4,860 (76.5) | 4,874 (76.3) | 4,376 (74.6) | 4,084 (72) | 3,469 (69.9) | -2.0 (-2.9 to -1.2)^*^ |
| CR (95% CI) | 773.8 (752.2-795.4) | 765 (743.5-786.5) | 763 (741.6-784.4) | 746 (723.9-768.1) | 719.6 (697.6-741.7) | 698.8 (675.6-722.1) | -2.0 (-2.9 to -1.2)^*^ |
| Untreated^b^ |  |  |  |  |  |  |  |
| No. (%) | 1,443 (22.6) | 1,493 (23.5) | 1,514 (23.7) | 1,490 (25.4) | 1,591 (28) | 1,495 (30.1) | 6.0 (3.7 to 8.2)^*^ |
| CR (95% CI) | 226.2 (214.5-237.8) | 235 (223.1-246.9) | 237 (225.1-248.9) | 254 (241.1-266.9) | 280.4 (266.6-294.1) | 301.2 (285.9-316.4) | 6.0 (3.7 to 8.3)^*^ |
| High income^f^ |  |  |  |  |  |  |  |
| Treated^a^ |  |  |  |  |  |  |  |
| No. (%) | 5,133 (76.7) | 5,005 (75.3) | 5,014 (73.4) | 4,783 (72.4) | 4,357 (69.8) | 3,882 (69.5) | -2.1 (-2.5 to -1.6)^*^ |
| CR (95% CI) | 767.1 (746.2-788.1) | 752.9 (732-773.7) | 734.3 (714-754.7) | 724.3 (703.7-744.8) | 697.6 (676.9-718.3) | 694.8 (673-716.7) | -2.1 (-2.5 to -1.6)^*^ |
| Untreated^b^ |  |  |  |  |  |  |  |
| No. (%) | 1,558 (23.3) | 1,643 (24.7) | 1,814 (26.6) | 1,821 (27.6) | 1,889 (30.2) | 1,705 (30.5) | 5.8 (4.5 to 7.2)^*^ |
| CR (95% CI) | 232.9 (221.3-244.4) | 247.1 (235.2-259.1) | 265.7 (253.4-277.9) | 275.7 (263.1-288.4) | 302.4 (288.8-316.1) | 305.2 (290.7-319.7) | 5.9 (4.5 to 7.2)^*^ |
| Low income^g^ |  |  |  |  |  |  |  |
| Treated^a^ |  |  |  |  |  |  |  |
| No. (%) | 2,658 (76.8) | 2,700 (74.9) | 2,684 (74.6) | 2,500 (73) | 2,365 (71.5) | 1,947 (66.3) | -2.5 (-3.9 to -1.1)^*^ |
| CR (95% CI) | 768.4 (739.2-797.6) | 749 (720.7-777.2) | 746.4 (718.1-774.6) | 729.7 (701.1-758.3) | 714.5 (685.7-743.3) | 663.1 (633.7-692.6) | -2.5 (-3.9 to -1.1)^*^ |
| Untreated^b^ |  |  |  |  |  |  |  |
| No. (%) | 801 (23.2) | 905 (25.1) | 912 (25.4) | 926 (27) | 945 (28.5) | 989 (33.7) | 6.8 (3.7 to 10.0)^*^ |
| CR (95% CI) | 231.6 (215.5-247.6) | 251 (234.7-267.4) | 253.6 (237.2-270.1) | 270.3 (252.9-287.7) | 285.5 (267.3-303.7) | 336.9 (315.9-357.8) | 6.9 (3.8 to 10.0)^*^ |
| High comorbidity^h^ |  |  |  |  |  |  |  |
| Treated^a^ |  |  |  |  |  |  |  |
| No. (%) | 9,556 (77.3) | 10,073 (76.1) | 10,542 (75.1) | 10,142 (73.3) | 9,736 (72) | 8,440 (69.3) | -2.1 (-2.6 to -1.6)^*^ |
| CR (95% CI) | 773.4 (757.9-788.9) | 760.6 (745.7-775.4) | 751 (736.7-765.4) | 733 (718.7-747.2) | 720.3 (706-734.6) | 693.1 (678.3-707.9) | -2.1 (-2.6 to -1.6)^*^ |
| Untreated^b^ |  |  |  |  |  |  |  |
| No. (%) | 2,800 (22.7) | 3,171 (23.9) | 3,495 (24.9) | 3,695 (26.7) | 3,780 (28) | 3,737 (30.7) | 6.0 (5.0 to 7.1)^*^ |
| CR (95% CI) | 226.6 (218.2-235) | 239.4 (231.1-247.8) | 249 (240.7-257.2) | 267 (258.4-275.6) | 279.7 (270.8-288.6) | 306.9 (297.1-316.7) | 6.0 (5.0 to 7.1)^*^ |
| Low comorbidity^i^ |  |  |  |  |  |  |  |
| Treated^a^ |  |  |  |  |  |  |  |
| No. (%) | 4,207 (78.7) | 3,539 (76.6) | 3,248 (75.8) | 2,849 (76) | 2,386 (71.5) | 1,815 (70.3) | -2.2 (-3.2 to -1.1)^*^ |
| CR (95% CI) | 787.2 (763.4-811) | 766.3 (741.1-791.6) | 758.3 (732.3-784.4) | 759.9 (732-787.8) | 714.8 (686.1-743.5) | 702.7 (670.3-735) | -2.2 (-3.2 to -1.1)^*^ |
| Untreated^b^ |  |  |  |  |  |  |  |
| No. (%) | 1,137 (21.3) | 1,079 (23.4) | 1,035 (24.2) | 900 (24) | 952 (28.5) | 768 (29.7) | 6.6 (3.6 to 9.7)^*^ |
| CR (95% CI) | 212.8 (200.4-225.1) | 233.7 (219.7-247.6) | 241.7 (226.9-256.4) | 240.1 (224.4-255.7) | 285.2 (267.1-303.3) | 297.3 (276.3-318.4) | 6.7 (3.7 to 9.8)^*^ |

NOTE: Data presented above include patients with five types of non-sex-specific cancer (gastric, colorectal, liver, pancreas, and lung) who were newly diagnosed with stage IV cancer from 2012 to 2017 and deceased between 2012 and 2018.Selection criteria for these non-sex-specific cancers was cancer mortality from the KCCR report. All values are presented as no.(%) per annum except for absolute difference and change in trend (APC with 95% CI)

Abbreviations: no., number; CR, crude rate; CI, confidence interval; APC, annual percent change; CI, confidence interval; y, years; KCCR, Korea Central Cancer Registry; CCI, Charlson comorbidity index

^a^Patients with newly diagnosed stage IV cancer who underwent surgery, chemotherapy, radiotherapy or combination treatment prior to death

^b^Patients with newly diagnosed stage IV cancer who did not receive any type of treatment for cancer prior to death

^c^Patients aged below 70 years (<70 years)

^d^Patients aged 70 years or older (≥70 years)

^e^Residing in the urban (capital/metropolitan) and rural area(city/town) defined by the administrative divisions in the Republic of Korea

^f^Highest quartile (quartile 4) based on the insurance premium (proxy for individual income status)

^g^Lowest quartile (quartile 1) based on the insurance premium (proxy for individual income status)

^h^Defined as CCI≥2 (without accounting for assigned weights in comorbid conditions related to cancer)

^i^Defined as CCI=1 or none (without accounting for assigned weights in comorbid conditions related to cancer)

**p*-value <0.05
